# Implementation of Spiegler–Kedem and Steric Hindrance Pore Models for Analyzing Nanofiltration Membrane Performance for Smart Water Production

**DOI:** 10.3390/membranes8030078

**Published:** 2018-09-06

**Authors:** Remya R. Nair, Evgenia Protasova, Skule Strand, Torleiv Bilstad

**Affiliations:** 1 Department of Chemistry, Bioscience and Environmental Engineering, University of Stavanger, Kjell Arholmsgate 41, 4036 Stavanger, Norway; evgy.pro@gmail.com (E.P.); torleiv.bilstad@uis.no (T.B.); 2 Department of Energy and Petroleum Engineering, University of Stavanger, Kjell Arholmsgate 41, 4036 Stavanger, Norway; skule.strand@uis.no

**Keywords:** nanofiltration, Spiegler–Kedem model, steric hindrance pore model, ion rejection, reflection coefficient, solute permeability, pure water permeability

## Abstract

A predictive model correlating the parameters in the mass transfer-based model Spiegler–Kedem to the pure water permeability is presented in this research, which helps to select porous polyamide membranes for enhanced oil recovery (EOR) applications. Using the experimentally obtained values of flux and rejection, the reflection coefficient *σ* and solute permeability *P_s_* have been estimated as the mass transfer-based model parameters for individual ions in seawater. The reflection coefficient and solute permeability determined were correlated with the pure water permeability of a membrane, which is related to the structural parameters of a membrane. The novelty of this research is the development of a model that consolidates the various complex mechanisms in the mass transfer of ions through the membrane to an empirical correlation for a given feed concentration and membrane type. These correlations were later used to predict ion rejections of any polyamide membrane with a known pure water permeability and flux with seawater as a feed that aids in the selection of suitable nanofiltration (NF) for smart water production.

## 1. Introduction

Nanofiltration (NF) membranes are pressure driven and selectively separate ions from mixed electrolyte solutes with low energy requirements compared to other desalination technologies. Smart water can be produced by modifying the ionic composition of seawater [1]. Smart water for EOR in carbonate and sandstone reservoirs require different ionic compositions depending on reservoir properties. Divalent ion-rich brine is required for carbonates, whereas a salinity of less than 5000 ppm is preferred for sandstones [1]. Production of smart water from seawater using membranes and the resulting power consumption was discussed in detail in our previous research [2]. However, selection of suitable membranes for smart water production is an extensive process. Thus, predicting membrane ion rejection limited to a couple of steps will avoid intensive membrane experiments.

Application of mathematical models to predict NF membrane performance for selective ion rejection is important for the optimal design and operation of NF membranes for smart water production. However, most modeling studies to date have considered only very dilute solutions and typically containing two or three types of ions. Modeling of concentrated solutions with multi-feed ions, such as seawater, predicts NF performance realistically with regard to industrial applications.

Spiegler–Kedem is a mass transfer-based model that relates flux to the concentration difference of a solute for a given membrane and solvent properties. The experimental data of flux versus rejection for individual ions for different membranes is used to validate a model. The model is developed using the estimated equation parameters or transport parameters in the Spiegler–Kedem model and is correlated to the structural parameters of a membrane using a steric hindrance pore model. This approach simplifies membrane performance prediction for a given feed ionic composition and provides a consolidated approach to various interacting phenomena that are difficult to define mathematically for mass transport. For the correlations predicted in this research, the model fitting is carried out for a given feed concentration with a certain membrane type (polyamide) so that active mechanisms for all the membranes are similar and can be easily understood. The proposed correlations can be used for predicting ion rejection, thereby aiding the selection of suitable NF membranes for smart water production administered to both carbonate and sandstone reservoirs.

The principal objective of this research is to develop a predictive model to quantify the selectivity of porous polyamide membranes with high feed concentrations for smart water production. To develop such a model, membrane transport parameters and effective pore size were determined using the Spiegler–Kedem model and a steric-hindrance pore model.

## 2. Theory

### 2.1. Nanofiltration Membranes

NF membranes permit preferential transport of ions. Separation processes are differentiated based on membrane pore sizes. NF membranes have pore sizes between 0.1 and 1 nm [3] with a molecular weight cut off (MWCO) of 100–5000 Da [4]. Mass transfer through NF includes convection and solution-diffusion [5]. NF selectively separates divalent and monovalent ions. This is mainly due to the strong dependence on the operating parameters, pressure, and feed concentrations, and on the membrane structural parameters such as pore radius and the ratio of membrane porosity to membrane thickness, *A_k_/Δx*. The separation mechanisms also depend on the hydrophilic/hydrophobic characteristics of the membrane [6].

The performance of the membranes is generally measured in terms of rejection *R* and flux *J_v_*. Rejection is a measure of the membrane’s ability to reject a solute. Membrane rejection is calculated using Equation (1).

(1) $$R=\left(1-\frac{C_{p}}{C_{f}}\right)$$

where *C_p_* and *C_f_* are the permeate and feed concentrations, respectively.

Flux *J_v_* (Lm^−2^ h^−1^) is calculated using Equation (2)

(2) $$J_{v}=\frac{V}{t\times A}$$

where *V* is the volume of the permeate collected in a given time interval *t*, and *A* is the membrane area.

### 2.2. Spiegler–Kedem Model

Transport of solutes through a charged membrane can be described using the principles of non-equilibrium thermodynamics where the membrane is considered a black box. This approach allows the membranes to be characterized in terms of only the reflection coefficient *σ* and solute permeability *P_s_*. In a two-component system consisting of solute and water with flux *J_v_*, the solute flux *J_s_* is related by three membrane coefficients [7]:

1. The hydraulic permeability *L_p_.*
2. The solute permeability *P_s_.*
3. The reflection coefficient *σ.*

The relation between *J_v_* and *J_s_* and the membrane coefficients is given by Equations (3) and (4) as introduced by Kedem and Katchalsky [8].

(3) $$J_{V}=L_{p}\left(\Delta Ρ-\sigma \Delta \pi \right)$$

(4) $$J_{s}=P_{s}\Delta C_{s}+\left(1-\sigma \right)J_{v}\;C_{m}$$

where Δ*C_s_* = *C_m_* − *C_p_*, and *C_m_* is the solute concentration at the membrane surface. Δ*P* is the pressure difference between the feed and permeate, and Δπ is the osmotic pressure difference of the two fluids. According to Equation (4), the solute flux is the sum of diffusive and convective terms. Transport of the solute by convection is due to an applied pressure gradient across the membrane. The concentration difference on the membrane side and the permeate results in transport by diffusion.

When a high concentration difference exists between the retentate and the permeate, the Spiegler–Kedem model can be used [5], as in this research. The solute permeability coefficient *P_s_* and reflection coefficient *σ* can be obtained by fitting experimental values of solute rejection versus flux, according to the Spiegler–Kedem model as represented by Equations (5) and (6).

(5) $$R_{\mathrm{obs}}=\sigma \;\frac{\left(1-F\right)}{1-\sigma F}$$

where

(6) $$F=\exp\;(-\frac{1-\sigma }{P_{s}}J_{v)}$$

*F* is a dimensionless parameter that depends on the reflection coefficient, solvent flux, and solute permeability coefficient. The reflection coefficient represents the rejection capability of a membrane. No rejection occurs when *σ* = 0 and 100% rejection occur when *σ* = 1 [9]. Also, *σ* can be considered to represent the maximum rejection at an infinite volume flux.

Permeability can be defined as the flux of a solute or solvent through the membrane per unit driving force. *P_s_* is the overall solute permeability coefficient.

The Spiegler–Kedem model is based on irreversible thermodynamics to describe transport when the membrane structure and transport mechanism within the membrane is not fully understood [10]. The Spiegler–Kedem model is generally applied when there are no electrostatic interactions between the solute and the membrane such as when the membrane is uncharged or when the solute is neutral. NF membranes are mostly negatively or positively charged. Many authors have used this model with charged NF membranes [6,11] and suggested that *σ* and *P_s_* depend on the effective membrane charge and concentration of the feed solution. The effect of membrane charge is, however, neglected in this research for analyzing membrane performance at high feed concentrations.

The following assumptions were made while using the Spiegler–Kedem model in this research:

(1) The driving forces are pressure and concentration gradients.
(2) The model predicts the transport of the solute and solvent through the membrane irrespective of the type of solute, charge, solvent, and membrane.
(3) Membrane fouling and membrane sensitivity towards chemicals such as chlorine, effects of temperature, and pH are not considered.

### 2.3. Steric Hindrance Pore Model (SHP)

Structural parameters of the membranes were estimated using the SHP model developed by Nakao and Kimura [12] for the separation of aqueous solutions of a single organic solute by ultrafiltration membranes and was later successfully used for NF membranes by researchers such as Wang et al. [13]. According to the model, transport of spherical ions through cylindrical pores hindered by frictional forces and the steric effect are considered. Following this model, the membrane parameters *σ* and *P_s_* are given as

(7) σ=1−SF {1+(169)q2}

(8) Ps=D×SD(AkΔx)

where

(9) $$S_{D}={\left(1-q\right)}^{2}$$

(10) $$S_{F}=2{\left(1-q\right)}^{2}-{\left(1-q\right)}^{4}$$

and

(11) q=rsrp

where *S_D_* and *S_F_* are the steric hindrance factors for diffusion and convection respectively. *D* is diffusivity, *A_k_*/Δ*x* is the ratio of membrane porosity to membrane thickness, *r_s_* is the Stokes radius of the solute, and *r_p_* is the pore radius. The Stokes radii used for calculations [14,15] are presented in Table 1.

The stability of membranes is usually tested to assure the reliability of the experiments. This is mainly performed by measuring the pure water permeability (*L_p_* = *J_v_*/Δ*P*) of the membranes. The pure water permeability *L_p_* is also expressed by Hagen–Poiseuille in the pore model and is defined as

(12) $$L_{P}=r_{p}^{2}\left(\frac{A_{k}}{\Delta x}\right)/8µ$$

where *µ* is the viscosity.

## 3. Experimental Methods

Experiments were performed with a lab-scale membrane unit consisting of low-pressure and high-pressure pumps, a pressure valve, a pressure gauge, and two prefilters with 20 µ and 5 µ pore size as pre-treatment units upstream of the NF. One membrane is operated at a time and the retentate and permeate were recirculated to a 100 L feed tank to retain identical feed concentrations. The experiments were performed at room temperature with pure water and seawater. The applied pressure across the membranes ranged from 9 bar to 18 bar. Three trials were performed for each membrane with both pure water and seawater as feed. Pre-filtered seawater used for membrane experiments had total dissolved solids (TDS) of 30,400 mg/L, conductivity of 47.5 mS/cm, and pH at 7.9.

Prior to the experiments, the membranes were washed with pure water to remove any membrane preservatives. Eight different membranes with spiral wound configurations from two manufacturers (Nitto Hydranautics, Oceanside, CA, USA and Dow Filmtec, Oceanside, CA, USA) were used for the experiments and the membrane characteristics are provided in Table 2. NF 270 and SR 90 were from Dow Filmtec while all other six membranes were from Nitto Hydranautics. These commercially available membranes were negatively charged since their surface layers were made of polyamide or sulfonated polysulphone.

Individual ion concentrations in the feed, permeate, and retentate was measured using ion chromatography (Dionex^TM^ ICS-5000+ DP, from Thermo Fisher Scientific, Waltham, MA, USA). TDS and conductivity were measured using a TDS meter VWR collection CO3100N and pH by VWR Phenomenal pH 1100 L (both from VWR International Limited, Leicestershire, UK) 

All membranes, except for HYDRApro 501, had a maximum operating temperature of 45 °C. For HYDRApro 501, the operating temperature was pressure dependent: 41 bar at 65 °C and 14 bar at 90 °C. Maximum operating pressure for the rest of the membranes ranged from 41–41.6 bar according to the manufacturers.

Pure water permeability (*L_p_*) was experimentally determined by plotting flux *J_v_* versus transmembrane pressure Δ*P* and is represented by Lm^−2^ h^−1^ bar^−1^. The slope corresponding to each linear line determined the pure water permeability [10]. The hydraulic properties of the studied membranes were analyzed by measuring water flux as a function of pressure. Membrane water permeability was evaluated after achieving a steady-state condition with stable flux after operating the membranes for about 30 min.

## 4. Results and Discussion

### 4.1. Pure Water Permeability

**Figure 1:**
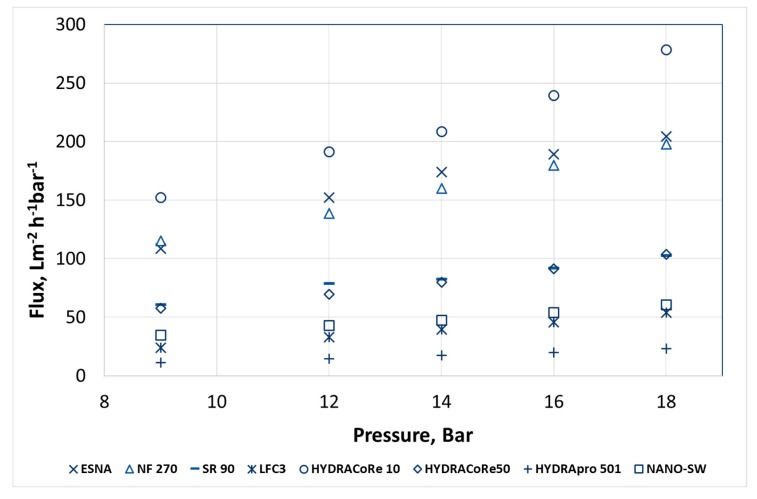
Pure water flux as a function of operating pressure for eight different membranes.

Figure 1 shows the dependency of operating pressure on flux through eight membranes. A linear relation was obtained for water flux as a function of operating pressure. According to Figure 1, the pure water permeability of the membranes decreased in the sequence HYDRACoRE 10 ˃ ESNA ˃ NF 270 ˃ HYDRACoRe 50 ˃ SR 90 ˃ NANO-SW ˃ LFC3 ˃ HYDRApro 501. 

LFC3 is a reverse osmosis membrane while HYDRApro 501 is used specifically for industrial applications with difficult feed streams, according to the manufactures. The permeabilities of these two membranes were lowest among the tested membranes. Thus, only pure water permeability experiments were performed for LFC3 and HYDRApro 501 membranes and these two membranes were not considered for further calculations of membrane transport parameters.

Relatively high flux was obtained for the other six membranes. High fluxes of these NF membranes at low pressure confirmed that NF membranes can be used as in energy saving compared to reverse osmosis membranes. Table 3 shows the water permeability of membranes when pure water and seawater were used as the feed.

*L_p_* of the tested membranes did not vary throughout the experiments. Hence, the membranes could be considered stable during the experimental period.

The effect of feed concentrations on the membrane flux was evident from the difference in water permeability between the two solutions in Table 3. Pure water permeability was highest through HYDRACoRe10, suggesting more open pores compared to the other tested membranes.

### 4.2. Calculation of σ, P_s_, and r_p_ Based on the Spiegler–Kedem and SHP Models

Experimental results for rejection and flux during permeation experiments with seawater were calculated using Equations (1) and (2). First, the transport parameters *σ* and *P_s_* for each ion were estimated using a nonlinear least squares method by fitting the Spiegler–Kedem model by plotting rejection versus flux for six membranes. Coefficients selected were with above 95% confidence bounds. Second, the pore radius based on individual ion rejection data for every membrane was determined from its membrane parameter *σ* based on the steric hindrance pore model (SHP) using Equations (7), (10), and (11). The value for *r_p_* (determined as = *r_s_*/*q*) were calculated using the Stokes radius of the solute (*r_s_*) as presented in Table 1.

**Figure 2:**
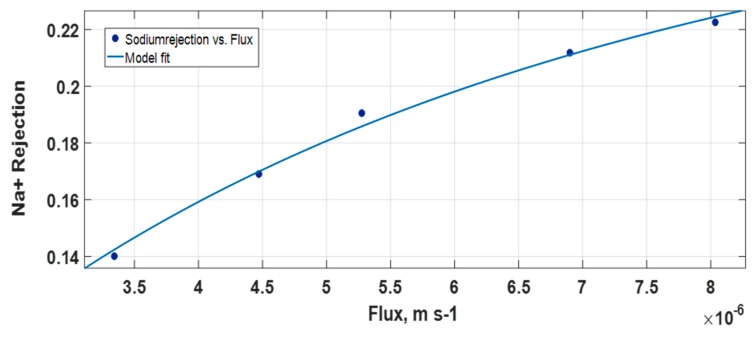
Rejection versus flux (m s^-1^) for Na^+^ for NANO-SW.

Membrane parameters were estimated by fitting rejection versus flux using the Spiegler–Kedem equation. Figure 2 shows the dependency of the real rejection on volume flux for Na^+^ for NANO-SW. The data points present the rejection values from the experiment and the solid line shows the values calculated using the Spiegler–Kedem equation with the best-fitted *σ* and *P_s_*. Figure 2 shows that the theoretical curves are in close agreement with experimental values.

The effective membrane pore radius for each ion was calculated from the transport parameters *σ* and *P_s_* based on the SHP model when seawater was used as the feed and is presented in Table 4.

Table 4 shows that reflection coefficients and solute permeability vary for each ion. The pore radii of these membranes were calculated using the Stokes radius of each ion. It was earlier reported by Luo and Wan [16] that the *r_p_* of NF 270 is 0.43 nm. The pore size of NF 270 was previously determined using atomic force microscopy by Hilal et al. [17] and suggested to be between 0.47–0.99 nm with a mean of 0.71 nm. An average pore size of 0.47 nm was determined for NF 270 using the SHP model in this research. The calculated pore size of NF 270 was in the same range as recorded by several researchers confirming the validity of the calculations. The results show that for these membranes, a pore size distribution was more likely than a fixed pore size, and the identification of an effective pore radius does not indicate the presence of geometrically defined pores in NF membranes.

According to Table 4, polyamide membranes showed better rejection for divalent ions since the reflection coefficient was high for divalent ions compared to monovalent ions. According to the obtained results, the Spiegler–Kedem model was able to fit the experimental data of flux versus rejection for all ions and for all membranes except for HYDRACoRe 10. For HYDRACoRe 10, negative Cl^−^ reflection coefficients were obtained for all performed trials with the model. This could be due to the very low rejection of Cl^−^ or probably a negative rejection of Cl^−^ even though it was not observed during experiments. Negative rejection implies that the system has more Cl^−^ in the permeate compared to the feed. Negative rejection of an ion occurs when a higher concentration of that ion is present in the smaller permeate volume relative to the larger feed volume. Negative rejection is observed mostly at low operating pressures [18]. The results show that HYDRACoRe 10 membrane has a larger pore size than the usual NF range which explains the poor ion separation of HYDRACoRe 10.

Table 4 shows that membranes with larger pore sizes had lower reflection coefficients. In other words, membranes with higher pure water permeability had lower individual ion reflection coefficients. A relative pore size comparison was performed with Mg^2+^ since it is a divalent cation with the highest Stokes radius compared to other ions tested for pore radius calculations, along with the fact that Mg^2+^ is attracted by the negatively charged membrane (unlike SO_4_^2−^) and would therefore permeate the membrane easily if the pore size was appropriately large for the ion. Hence, with respect to Mg^2+^, the pore size of the tested membranes was in the sequence HYDRACoRe 10 > ESNA > HYDRACoRe 50 > NF 270 > SR 90 > NANO-SW.

However, the high feed concentrations and the ionic interactions that occurred among unaccounted ions and major ions in seawater, along with the interactions between ions and the membrane, added to the overall complexity in separation mechanisms of NF membranes. This provides a challenge to any model based on high feed concentrations.

### 4.3. Selection of NF Membranes for Smart Water Production Using a Predictive Model

The ionic composition required for smart water depends mainly on the type of reservoir. For carbonate reservoirs, an NF membrane with a high rejection of divalent ions and low monovalent ion rejection should be selected. For sandstone reservoirs, low salinity is preferred. Thus, a membrane with moderate flux will be suitable, which results in low divalent ion permeation.

According to Equation (12), pure water permeability is a parameter that combines the structural properties of the membrane and is used as a critical parameter that determines the ion rejection of a membrane. The only other property that influences water permeability is the feed viscosity, as shown in Equation (12). During the experiments, the structural parameters remained the same provided temperature and pH of the feed are controlled. Several researchers [19,20] have established that temperature and pH affect the pore size and change the flux. In this research, the difference in viscosity between pure water and seawater was neglected when *L_p_* was used for correlating the reflection coefficient and solute permeability of membranes. 

Thus, according to Equation (12), pure water permeability was directly related to the structural parameters such as effective membrane pore radius, and to *A_k_/*Δ*x* (ratio of membrane porosity to membrane thickness). It can be inferred that the transport parameters of a solute are related to the structural properties of a specific membrane, as shown in Equations (7)–(11). Knowing the transport parameters, it is possible to predict the rejection (*R*_obs_) of a membrane using the Spiegler–Kedem model.

#### 4.3.1. Relating *L_p_* with *σ* and *P_s_*

*L_p_* versus *σ* and *P_s_* of individual ions were plotted to find a relation between pure water permeability, reflection coefficient, and *P_s_*. Transport parameters were calculated for four polyamide membranes, ESNA, NF 270, SR 90, and NANO-SW with varying *L_p_*. These four membranes were chosen since:

(1) Table 4 shows that HYDRACoRe 10 had poor ion separation. HYDRACoRe 50, made of sulfonated polyethersulfone, was not used to have comparable membrane materials for the model.
(2) The *L_p_* chosen for the plot to create the model was in the range required for smart water production. Pure water permeability higher than that of ESNA would have resulted in very low divalent ion rejection. Choosing a membrane with lower permeability than NANO-SW meant a tighter membrane leading to higher rejection for any flux and low recovery thereby increasing power consumption.

**Figure 3:**
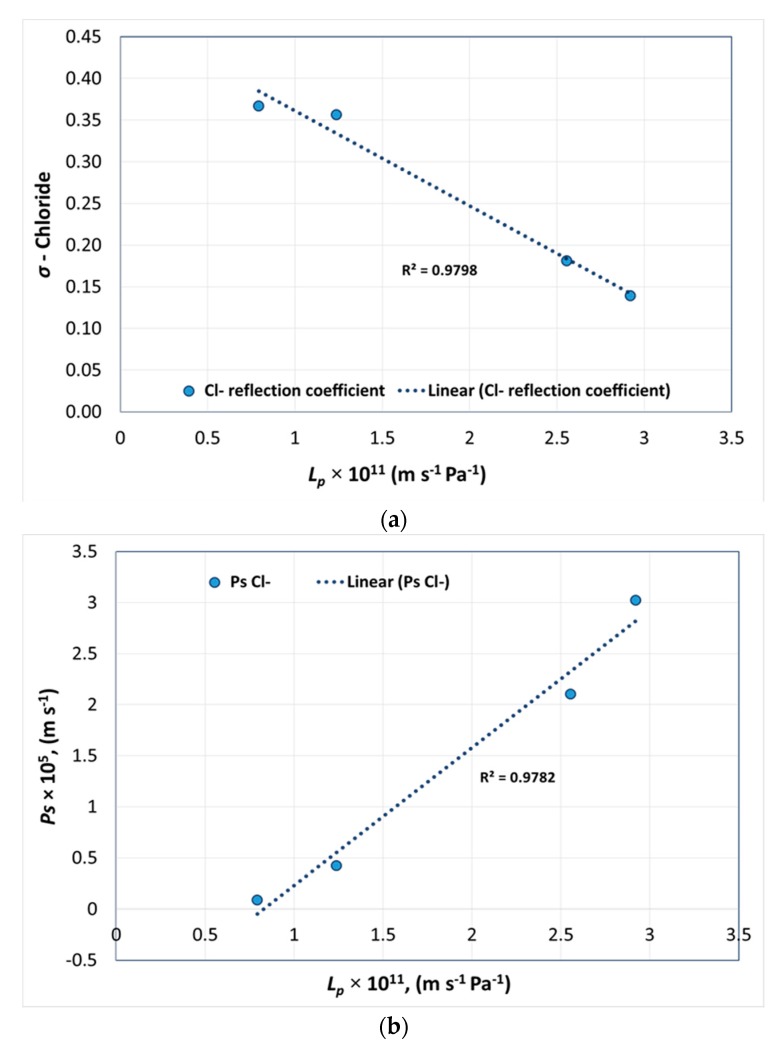
Pure water permeability versus (**a**) reflection coefficient and (**b**) solute permeability of chloride.

Figure 3a shows the pure water permeability of polyamide NF membranes versus *σ* and Figure 3b presents *L_p_* versus solute permeability *P_s_* of chloride for each membrane.

Figure 3a shows that with an increase in water permeability, the reflection coefficient of ions decreased whereas Figure 3b shows that the solute permeability increased. This confirmed that when the effective membrane pore radius increases, permeability increases, resulting in lower ion rejection. 

**Figure 4:**
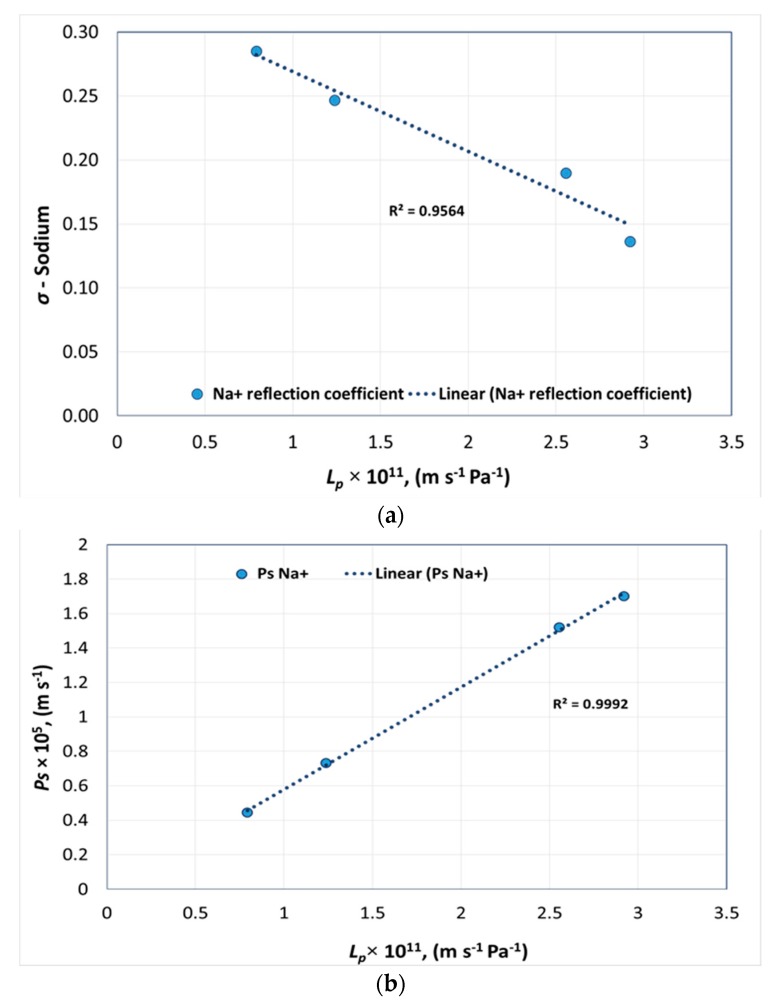
Pure water permeability versus (**a**) reflection coefficient and (**b**) solute permeability of sodium.

Similarly, Figure 4a and Figure 4b represents the pure water permeability of NF membranes versus *σ* and *P_s_* of sodium for each membrane, respectively.

**Figure 5:**
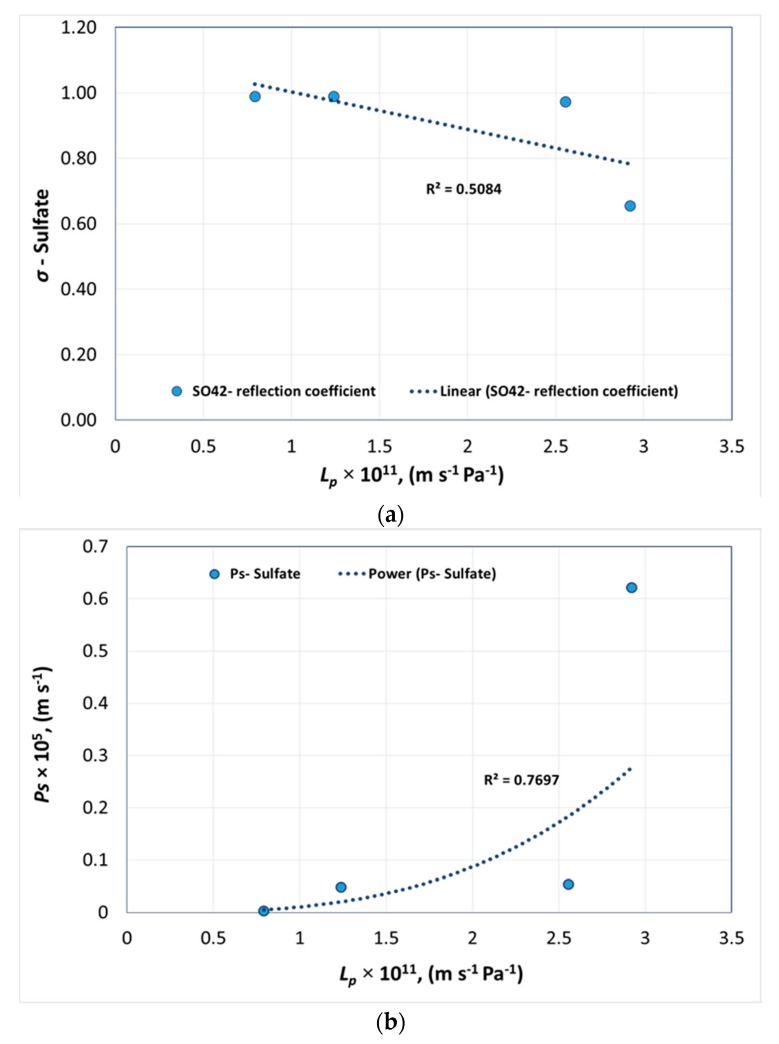
Pure water permeability versus (**a**) reflection coefficient and (**b**) solute permeability of sulfate.

Figure 5a presents the pure water permeability of membranes versus *σ* and Figure 5b presents *L_p_* versus *P_s_* of sulfate for each membrane.

According to Figure 5a, the sulfate reflection coefficient shows a sharp decline with a small change in water permeability. This was mainly because of divalent anion on the negatively charged membrane surface. In Figure 5b showing pure water permeability versus *P_s_*, the sulfate permeability remains unchanged for a range of permeabilities until approximately 2.6 × 10^−11^ m s^−1^ Pa^−1^. After this value, a sharp increase was observed similar to the sharp decline in reflection coefficient of sulfate. A deviation in the reflection coefficient and solute permeability of SO_4_^2−^ can be explained in relation to the thermodynamic properties of the ion. Ion permeation through a membrane is affected by the hydrated size and hydration free energy of the ions. During membrane transport, the transmembrane pressure creates shear stress that results in ions with low hydration energy being able to easily permeate through the membrane whereas ions with higher hydration energy and hydrated radius will be rejected by the membrane. SO_4_^2−^ is a divalent anion with a hydration free energy of −1145 KJ/mol and a hydrated radius of 0.379 nm [21]. When the negatively charged ion is in contact with a negatively charged membrane surface, ion repulsion occurs, resulting in a higher rejection. Similarly, to maintain electroneutrality on both sides of the membrane, anions with a lower hydration energy and hydrated radius permeate through the membrane. Hence, Cl^−^ will be preferentially permeated compared to SO_4_^2−^ due to a lower hydration energy of −340 KJ/mol and hydrated radius of 0.324 nm. In Figure 5a, for ESNA, the reflection coefficient for SO_4_^2−^ was lower at 0.66, whereas for the other three membranes, the SO_4_^2−^ reflection coefficient was greater than 0.95. This can be explained with regard to the *r_p_* calculated relative to Mg^2+^ as presented in Table 4. The pore radius *r_p_* calculated was 0.86 nm, thus SO_4_^2−^ permeated more for ESNA due to the steric effect resulting in lower *σ* and higher *P_s_* compared to the other three membranes with a pore size close to 0.4 nm that is in close proximity to the SO_4_^2−^ hydrated radius. Hence, a combination of steric effect and divalent anion-membrane repulsion prompted SO_4_^2−^ rejection in NANO-SW, SR 90, and NF 270.

**Figure 6:**
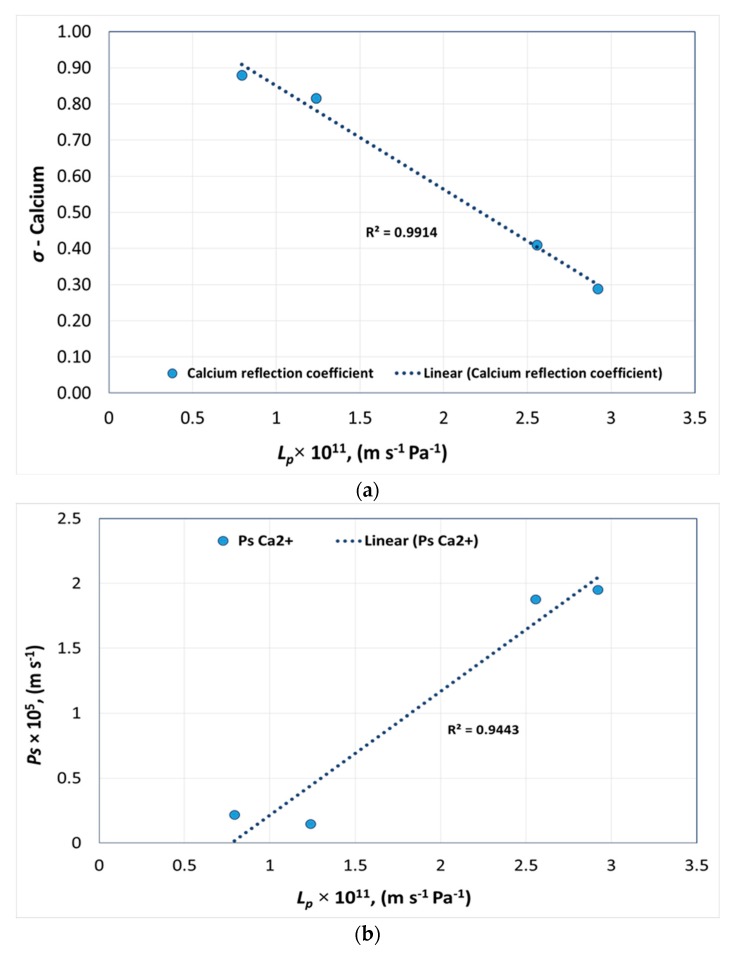
Pure water permeability versus (**a**) reflection coefficient and (**b**) solute permeability of calcium.

Figure 6a,b shows the pure water permeability of membranes versus *σ* and *P_s_* of calcium for each membrane, respectively.

According to Figure 6a, the reflection coefficient decreased gradually with increasing permeability. However, a small variation in calcium permeability was observed at lower permeabilities as shown in Figure 6b.

**Figure 7:**
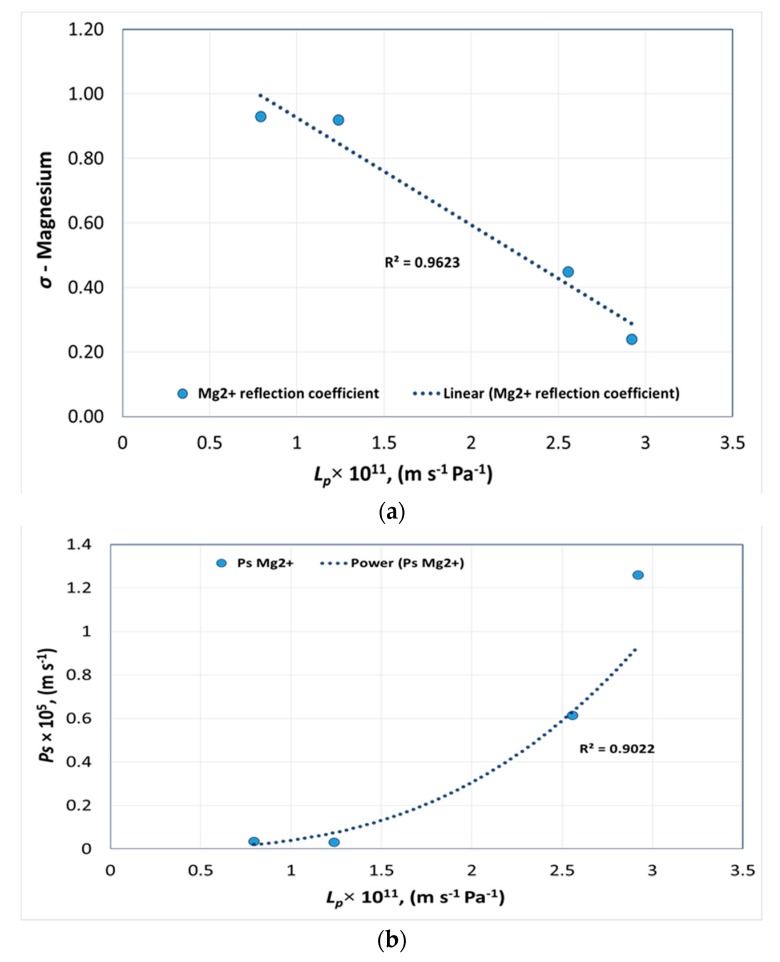
Pure water permeability versus (**a**) reflection coefficient and (**b**) solute permeability of magnesium.

Figure 7a,b shows the pure water permeability of membranes versus *σ* and *P_s_* of magnesium for each membrane, respectively.

According to Figure 7a, the reflection coefficient of Mg^2+^ deviated slightly from linear behavior for membranes with low pure water permeability. Mg^2+^ is a divalent cation with a hydration energy of −1922 KJ/mol with a hydrated radius of 0.470 nm [21]. According to Figure 7a,b, when pure water permeability decreased with respect to pore radius, the reflection coefficient of Mg^2+^ increased, confirming the higher rejection and lower permeation of Mg^2+^. The deviation from linear behavior was observed for membranes (NANO-SW and SR 90) with a calculated *r_p_* ≈ 0.4 nm with respect to Mg^2+^, where *r_p_* is close to its hydrated radius.

#### 4.3.2. Correlations for the Determination of *σ* and *P_s_* of a Polyamide Membranes

The correlation developed was considered valid if the feed is seawater with no change in ionic concentration and viscosity for all tested polyamide membranes.

The following equations were obtained from Figure 3, Figure 4, Figure 5, Figure 6 and Figure 7, to determine *σ* and *P_s_* of each ion with a given pure water permeability *L_p_*_0_.

(13) $$\sigma_{Cl^{-}}=-1\times 10^{10}\times L_{p0}+0.4749$$

(14) $$\sigma_{Na^{+}}=-6\times 10^{9}\times L_{p0}+0.3318$$

(15) $$\sigma_{SO_{4}{}^{2-}}=-1\times 10^{10}\times L_{p0}+1.118$$

(16) $$\sigma_{Ca^{2+}}=-3\times 10^{10}\times L_{p0}+1.1354$$

(17) $$\sigma_{Mg^{2+}}=-3\times 10^{10}\times L_{p0}+1.2559$$

(18) $$P_{s_{Cl^{-}}}=1\times 10^{11}\times L_{p0}-1.1144$$

(19) $$P_{s_{Na^{+}}}=6\times 10^{10}\times L_{p0}-0.0147$$

(20) $$P_{s_{SO_{4}{}^{2-}}}=4\times 10^{31}\times L_{p0}{}^{3.0496}$$

(21) $$P_{s_{Ca^{2+}}}=1\times 10^{11}\times L_{p0}-0.7388$$

(22) $$P_{s_{Mg^{2+}}}=9\times 10^{30}\times L_{p0}{}^{2.9414}$$

As previously explained, the correlations represented by Equations (13)–(22) are applicable only for seawater with similar TDS and ionic composition. For a change in feed, the coefficients need to be established through experimental data. Equations (13)–(22) can be used for determining *σ* and *P_s_* of polyamide membranes with pure water permeabilities between 5 × 10^−12^ to 3 × 10^−11^ m s^−1^ Pa^−1^, which include membranes with a pore size of 0.4 to 0.86 nm, according to Table 4. 

The following steps were performed to run the model for predicting transport parameters and rejection.

(1) Using Equations (13)–(22), the model was run to predict *σ*_theoretical_ and *P_s,_*_theoretical_ for two NF membranes with pure water permeabilities as in Table 5.
(2) Flux for the above-mentioned NF membranes with seawater as feed was calculated using Equation (2). A random flux value at 12 bar was chosen for the model.
(3) The values for *σ*_theoretical_ and *P_s_*_,theoretical_, and flux at 12 bar was substituted into Equations (5) and (6) to calculate the theoretical rejection (*R*_theoretical_).
(4) To validate the calculated equations, ion rejection by the two chosen NF membranes was experimentally determined (*R*_experimental_) using Equation (1) for individual ions in seawater. These rejection values were plotted against the respective membrane flux values, and transport parameters were determined by fitting the values using the Spiegler–Kedem equation. Hence, *σ*_experimental_ and *P_s,_*_experimental_ were determined.

Table 5 shows the results obtained based on the model and on experiments performed by two chosen NF membranes.

Table 5 shows a close correlation between the model and experimental values of *σ*, *P_s_*, and rejection of all ions except for Ca^2+^ for the membrane with lower pure water permeability. This validates the robustness of the model. Table 5 indicates that rejection for the divalent anion SO_4_^2−^ was highest for all tested membranes indicating the negative surface of the NF membranes. Focusing on the rejection of divalent cations, Mg^2+^ was rejected more than Ca^2+^ due to its larger Stokes radius as shown in Table 1.

The individual ion selectivity is a key parameter for selecting appropriate membrane for smart water production. In this research, the Spiegler–Kedem model was used for determining individual ion transport through the membrane rather than overall solute transport, which has been extensively studied previously. The study is relevant for end users to select proper NF membranes for producing smart water without extensive membrane experiments.

## 5. Conclusions

Membrane transport parameters were determined by fitting the Spiegler–Kedem equation using flux and rejection values obtained from experiments using six NF membranes. The theoretical rejection values obtained by fitting the Spiegler–Kedem equation showed good correlations with experimental values for NF membranes with a similar membrane material. It was evident that it was difficult to increase the membrane water flux without losing ion selectivity and membrane flux was directly related to the effective membrane pore radius. The flux was higher for membranes with *r_p_* > 0.7 nm. However, membrane ion rejection decreased with higher *r_p_*. The hypothetical pore radii of six membranes were evaluated from permeation experiments with charged ions using a steric hindrance pore model. The pore radii of membranes were estimated from 0.4 nm to 2.15 nm. The experiments concluded that the membranes had a pore size distribution rather than a single pore radius. A sharp change in *σ* and *P_s_* of sulfate were observed when plotted against pure water permeabilities of polyamide membranes. Hence, choosing an NF membrane for smart water production in carbonates requires much attention when having pure water permeabilities above 2.6 × 10^−11^ m s^−1^ Pa^−1^ where the SO_4_^2−^ rejection will be low. The suggested method helps to predict NF rejection for smart water production from seawater and for feeds with a high concentration and multi-ionic solutions as in softening and desalination.

## Figures and Tables

**Table 1:** Stokes radii of major ions used for calculations [14,15].

Ions	Cl^−^	Na^+^	SO_4_^2−^	Ca^2+^	Mg^2+^
Stokes Radius (nm)	0.121	0.184	0.231	0.310	0.348

**Table 2:** Membrane characteristics as provided by the suppliers.

Membranes	HYDRACoRe10	HYDRACoRe50	NF 270	SR 90	ESNA	NANO-SW	LFC3	HYDRApro501
Material	Sulphonated Polyethersulfone		Composite Polyamide					
pH range	2–11		3–10		2–10	3–9	2–10.6	2–11
Area (m^2^)	2.3		2.6		2.3			

MWCO of HYDRACoRe10 and HYDRACoRe50 are 3000 and 1000 Daltons, respectively.

**Table 3:** The permeability of membranes with different feed solutions.

Membranes	Pure Water (L m^−2^ h^−1^ bar^−1^)	Seawater (L m^−2^ h^−1^ bar^−1^)
HYDRACoRe 10	13.56	9.5
ESNA	10.52	7.9
NF 270	9.38	6.1
HYDRACoRe 50	5.15	3.8
SR 90	4.46	3.3
NANO-SW	3.27	1.9
LFC3	2.85	-
HYDRApro 501	1.32	-

**Table 4:** Calculated σ, *P_s_*, and average *r_p_* for ions for all tested membranes.

Membranes	Ions	*σ* (−)	*P_s_* (m s^−1^)	q	*r_p_* (nm)
ESNA	Cl^−^	0.14	3.023 × 10^−5^	0.30	0.41
	Na^+^	0.14	1.701 × 10^−5^	0.29	0.63
	SO_4_^2−^	0.66	6.211 × 10^−6^	0.69	0.34
	Ca^2+^	0.29	1.953 × 10^−5^	0.44	0.71
	Mg^2+^	0.24	1.26 × 10^−5^	0.40	0.86
NF 270	Cl^−^	0.18	2.105 × 10^−5^	0.34	0.35
	Na^+^	0.19	1.521 × 10^−6^	0.35	0.52
	SO_4_^2−^	0.97	5.341 × 10^−7^	0.93	0.25
	Ca^2+^	0.41	1.879 × 10^−5^	0.53	0.58
	Mg^2+^	0.45	6.154 × 10^−6^	0.56	0.62
SR 90	Cl^−^	0.36	4.241 × 10^−6^	0.50	0.24
	Na^+^	0.25	7.313 × 10^−6^	0.41	0.45
	SO_4_^2−^	0.99	4.859 × 10^−7^	0.96	0.24
	Ca^2+^	0.82	1.474 × 10^−6^	0.79	0.39
	Mg^2+^	0.92	3.276 × 10^−7^	0.85	0.41
HYDRACoRe10	Cl^−^	−0.01	−4.844 × 10^−7^	-	-
	Na^+^	0.03	3.115 × 10^−5^	0.13	1.42
	SO_4_^2−^	0.16	1.728 × 10^−5^	0.32	0.73
	Ca^2+^	0.15	7.254 × 10^−5^	0.31	0.99
	Mg^2+^	0.05	5.447 × 10^−5^	0.16	2.15
HYDRACoRe50	Cl^−^	0.17	1.329 × 10^−5^	0.33	0.37
	Na^+^	0.24	1.538 × 10^−5^	0.40	0.46
	SO_4_^2−^	0.67	3.849 × 10^−6^	0.70	0.33
	Ca^2+^	0.32	5.928 × 10^−6^	0.47	0.67
	Mg^2+^	0.38	1.417 × 10^−5^	0.51	0.68
NANO-SW	Cl^−^	0.37	9.045 × 10^−7^	0.50	0.24
	Na^+^	0.29	4.439 × 10^−6^	0.44	0.42
	SO_4_^2−^	0.99	3.298 × 10^−8^	0.96	0.24
	Ca^2+^	0.88	2.171 × 10^−6^	0.84	0.37
	Mg^2+^	0.93	3.471 × 10^−7^	0.88	0.40

**Table 5:** Comparison of experimental and theoretical values from the Spiegler–Kedem equation.

Pure Water Permeability, m s^−1^ Pa^−1^	Flux at 12 bar, m s^−1^	Ions	*σ*_theoretical_	*σ*_experimental_	*P_s,_*_theoretical_, m s^−1^	*P_s,_*_experimental_, m s^−1^	*R*_theoretical_	*R*_experimental_
2.56 × 10^−11^	2.06 × 10^−5^	Cl^−^	0.22	0.18	1.44 × 10^−5^	2.11 × 10^−5^	0.16	0.11
		Na^+^	0.18	0.19	1.52 × 10^−5^	1.52 × 10^−5^	0.13	0.14
		SO_4_^2−^	0.83	0.97	1.99 × 10^−6^	5.34 × 10^−7^	0.79	0.96
		Ca^2+^	0.37	0.41	1.82 × 10^−5^	1.88 × 10^−5^	0.23	0.24
		Mg^2+^	0.44	0.45	6.27 × 10^−6^	6.15 × 10^−6^	0.42	0.41
1.24 × 10^−11^	8.90 × 10^−6^	Cl^−^	0.35	0.36	1.23 × 10^−6^	4.24 × 10^−6^	0.35	0.29
		Na^+^	0.26	0.25	7.28 × 10^−6^	7.31 × 10^−6^	0.17	0.16
		SO_4_^2−^	0.99	0.99	2.18 × 10^−7^	4.86 × 10^−7^	0.97	1.00
		Ca^2+^	0.76	0.82	4.99 × 10^−6^	1.47 × 10^−6^	0.53	0.75
		Mg^2+^	0.89	0.92	7.44 × 10^−7^	3.28 × 10^−7^	0.85	0.96

## Supplementary Materials

The following are available online at http://www.mdpi.com/2077-0375/8/3/78/s1, Figure S1: Rejection versus flux for Na^+^ for ESNA fitted using Spiegler–Kedem model, Figure S2: Rejection versus flux for Na^+^ for HYDRACoRe10 fitted using Spiegler–Kedem model, Figure S3: Rejection versus flux for Na^+^ for HYDRACoRe50, Figure S4: Rejection versus flux for Na^+^ for NF270, Figure S5: Rejection versus flux for Na^+^ for SR 90, Figure S6: Rejection versus flux for Na^+^ for NANO SW, Figure S7: Rejection versus flux for Na^+^ for all NF membranes.
