# Supplementary material for: Implementation of Spiegler–Kedem and Steric Hindrance Pore Models for Analyzing Nanofiltration Membrane Performance for Smart Water Production
Source: Membranes (Basel). 2018 Sep 6;8(3):78. doi: 10.3390/membranes8030078 (PMC6160980; doi:10.3390/membranes8030078)
Supplement: Supplementary file 1 [file membranes-08-00078-s001.pdf]

membranes

Supplementary Materials

Implementation of Spiegler–Kedem and Steric Hindrance Pore Models for Analyzing Nanofiltration Membrane Performance for Smart Water Production

Remya R. Nair [1,*], Evgenia Protasova [1], Skule Strand [2] and Torleiv Bilstad [1]

[1] Department of Chemistry, Bioscience and Environmental Engineering, University of Stavanger, Kjell Arholmsgate 41, 4036 Stavanger, Norway; evgy.pro@gmail.com (E.P.); torleiv.bilstad@uis.no (T.B.)
[2] Department of Energy and Petroleum Engineering, University of Stavanger, Kjell Arholmsgate 41, 4036 Stavanger, Norway; skule.strand@uis.no
* Correspondence: remya.nair@uis.no

Received: 8 August 2018; Accepted: 31 August 2018; Published: date

Sodium Rejection Versus Flux for Six Nf Membranes Fitted with Spiegler–Kedem Model

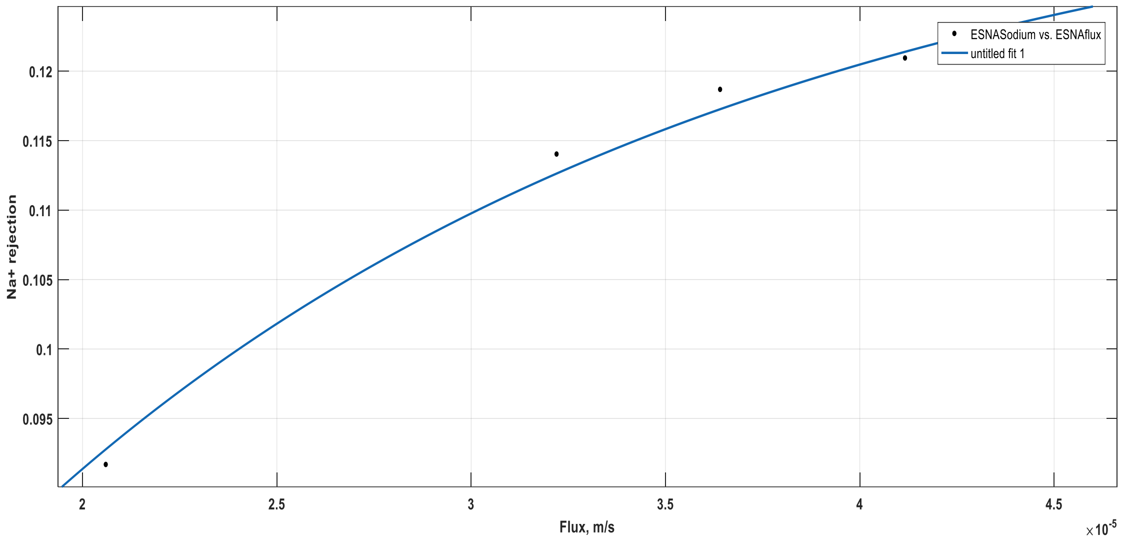

Figure S1. Rejection versus flux for Na^{+} for ESNA fitted using Spiegler–Kedem model.

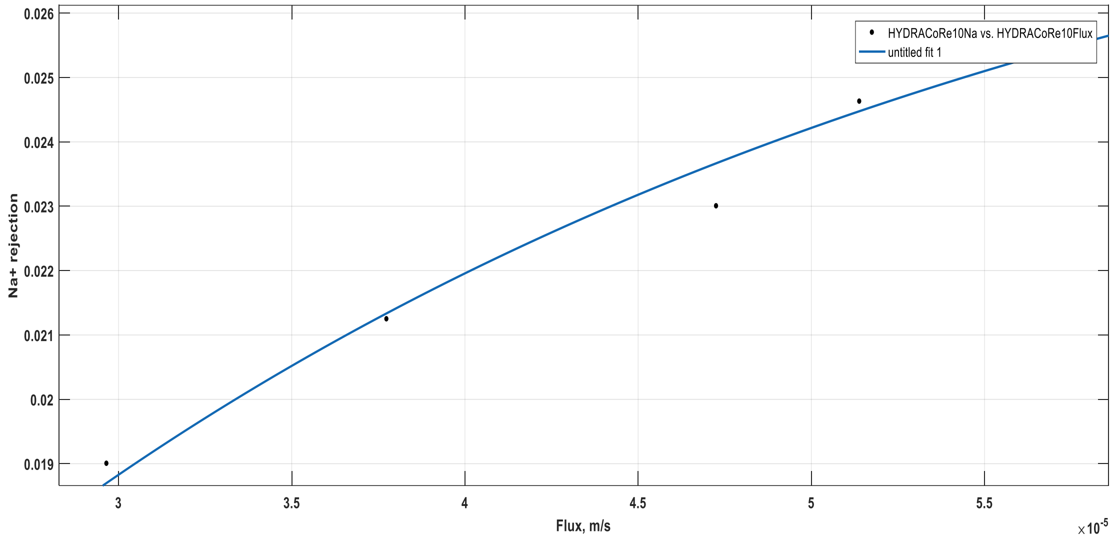

Figure S2. Rejection versus flux for Na^{+} for HYDRACoRe10 fitted using Spiegler–Kedem model.

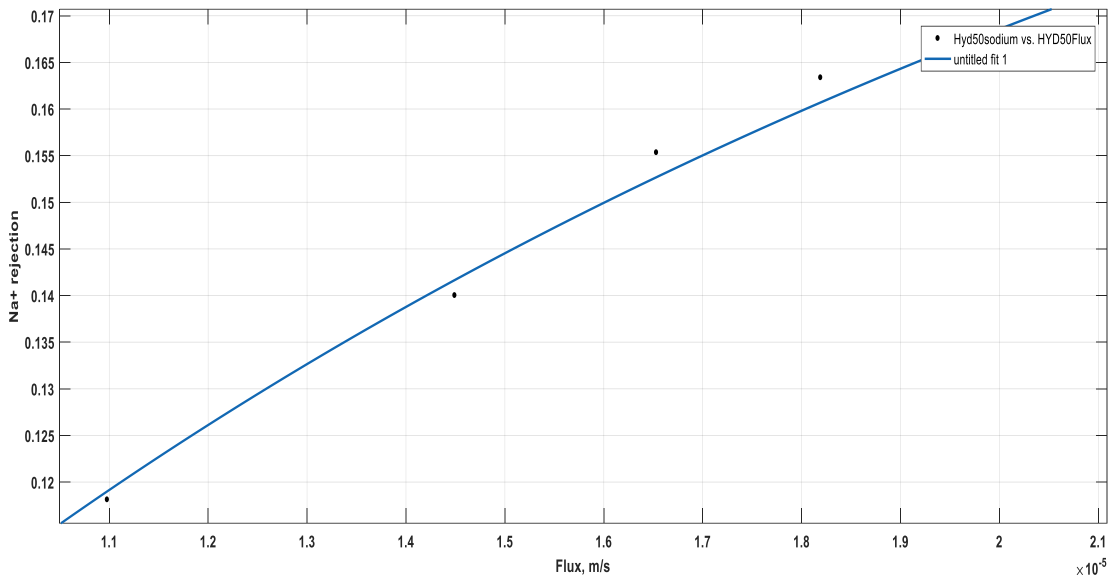

Figure S3. Rejection versus flux for Na^+ for HYDRACoRe50.

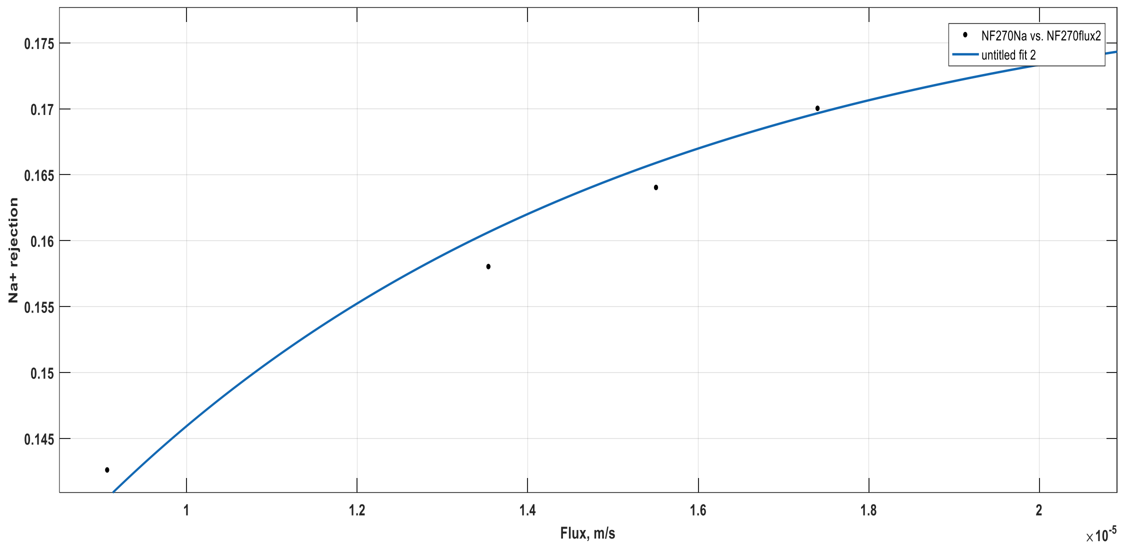

Figure S4. Rejection versus flux for Na^+ for NF270.

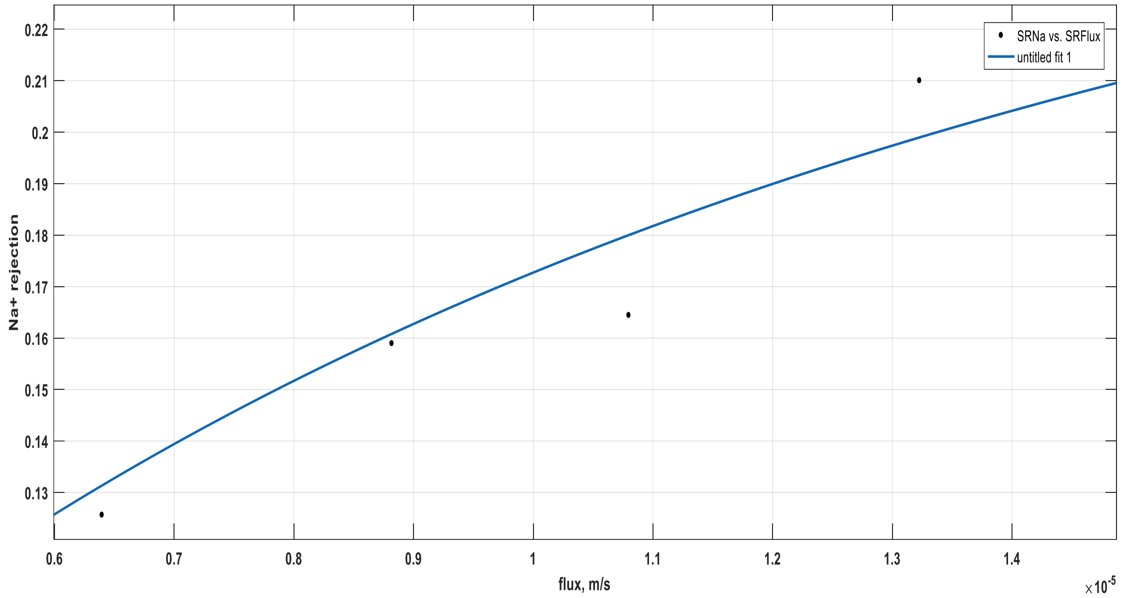

Fig

Figure S5. Rejection versus flux for Na^{+} for SR 90

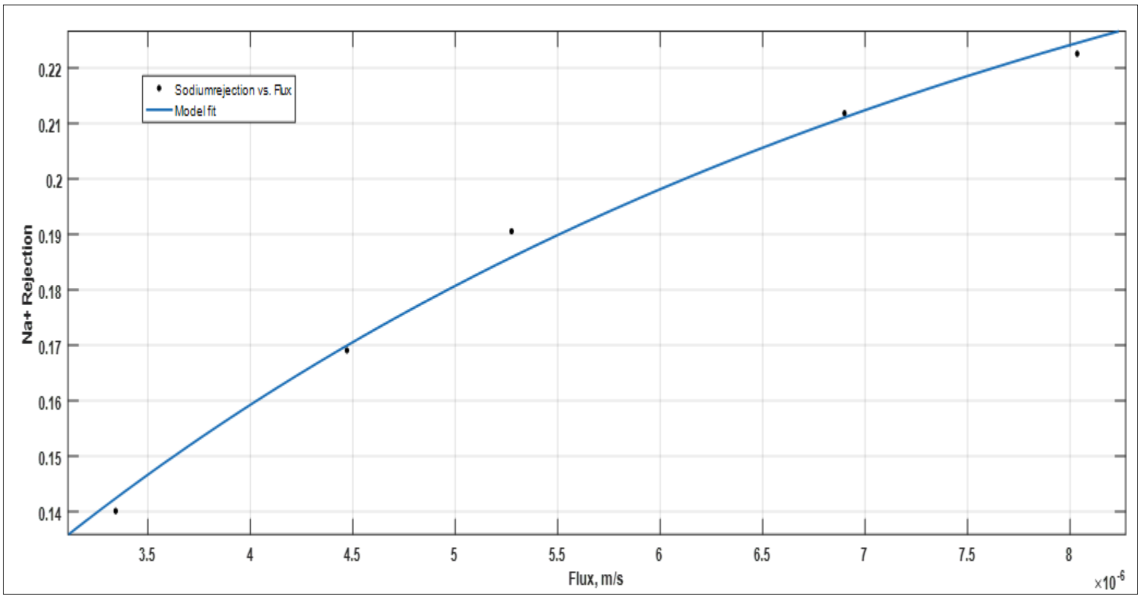

Figure S6. Rejection versus flux for Na^{+} for NANO SW.

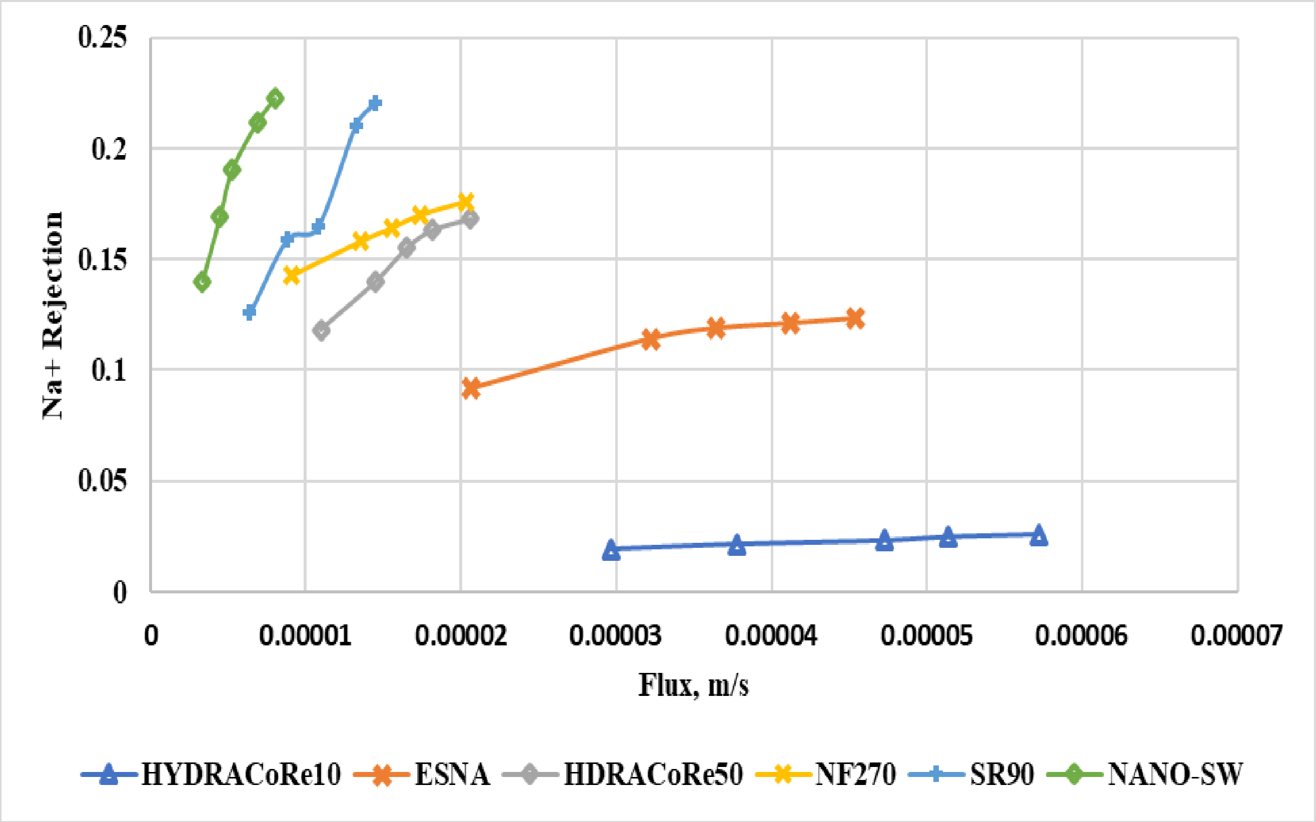

Figure S7. Rejection versus flux for Na^{+} for all NF membranes.
